# Functional characterisation of obesity-associated *MRAP2* variants on MC4R and GHSR signalling

**DOI:** 10.1093/hmg/ddag010

**Published:** 2026-02-27

**Authors:** Alejandra V Rodríguez Rondón, Karina Prins, Femke Volker, Eline E P L van der Walle, Cornelis J de Groot, Erica L T van der Akker, Elisabeth F C van Rossum, Mieke M van Haelst, Patric J D Delhanty, Jenny A Visser

**Affiliations:** Obesity Centre CGG, Erasmus MC, University Medical Center Rotterdam, P.O. Box 2040, 3000 CA Rotterdam, the Netherlands; Department of Internal Medicine, Erasmus MC, University Medical Center Rotterdam, P.O. Box 2040, 3000 CA Rotterdam, the Netherlands; Obesity Centre CGG, Erasmus MC, University Medical Center Rotterdam, P.O. Box 2040, 3000 CA Rotterdam, the Netherlands; Department of Internal Medicine, Erasmus MC, University Medical Center Rotterdam, P.O. Box 2040, 3000 CA Rotterdam, the Netherlands; Obesity Centre CGG, Erasmus MC, University Medical Center Rotterdam, P.O. Box 2040, 3000 CA Rotterdam, the Netherlands; Department of Internal Medicine, Erasmus MC, University Medical Center Rotterdam, P.O. Box 2040, 3000 CA Rotterdam, the Netherlands; Obesity Centre CGG, Erasmus MC, University Medical Center Rotterdam, P.O. Box 2040, 3000 CA Rotterdam, the Netherlands; Department of Paediatrics, Division of Endocrinology, Erasmus MC-Sophia Children’s Hospital, University Medical Center Rotterdam, P.O. Box 2040, 3000 CA Rotterdam, the Netherlands; Department of Paediatrics, Division of Endocrinology, Erasmus MC-Sophia Children’s Hospital, University Medical Center Rotterdam, P.O. Box 2040, 3000 CA Rotterdam, the Netherlands; Obesity Centre CGG, Erasmus MC, University Medical Center Rotterdam, P.O. Box 2040, 3000 CA Rotterdam, the Netherlands; Department of Paediatrics, Division of Endocrinology, Erasmus MC-Sophia Children’s Hospital, University Medical Center Rotterdam, P.O. Box 2040, 3000 CA Rotterdam, the Netherlands; Obesity Centre CGG, Erasmus MC, University Medical Center Rotterdam, P.O. Box 2040, 3000 CA Rotterdam, the Netherlands; Department of Internal Medicine, Erasmus MC, University Medical Center Rotterdam, P.O. Box 2040, 3000 CA Rotterdam, the Netherlands; Section Clinical Genetics, Department of Human Genetics, Amsterdam University Medical Center, Meibergdreef 9, 1105 AZ Amsterdam, the Netherlands; Obesity Centre CGG, Erasmus MC, University Medical Center Rotterdam, P.O. Box 2040, 3000 CA Rotterdam, the Netherlands; Department of Internal Medicine, Erasmus MC, University Medical Center Rotterdam, P.O. Box 2040, 3000 CA Rotterdam, the Netherlands; Obesity Centre CGG, Erasmus MC, University Medical Center Rotterdam, P.O. Box 2040, 3000 CA Rotterdam, the Netherlands; Department of Internal Medicine, Erasmus MC, University Medical Center Rotterdam, P.O. Box 2040, 3000 CA Rotterdam, the Netherlands

**Keywords:** Obesity, GPCR accessory protein, MRAP2 variants, GPCR signalling

## Abstract

Melanocortin-2 receptor accessory protein-2 (MRAP2) modulates the activity of hypothalamic melanocortin-4 (MC4R) and growth hormone-secretagogue (GHSR) receptors, which suppress and promote appetite, respectively. We investigate whether obesity-associated variants of MRAP2 alter their ability to modulate MC4R and GHSR signalling as a possible mechanistic link to the development of obesity. Functional effects of five obesity-associated *MRAP2* variants were analysed in HEK293 cells by co-expressing wild-type or variant MRAP2 with MC4R or GHSR. Endpoints included cell-surface and total expression, and ligand-induced second-messenger responses, β-arrestin-2 recruitment, and alternative G-protein activation. MRAP2 decreased basal MC4R cell-surface expression while GHSR cell-surface expression was not affected. In MC4R/MRAP2 expressing cells, maximal α-MSH-induced cAMP and β-arrestin-2 recruitment responses were increased. Similarly, ghrelin-induced Ca^2+^-mobilization in GHSR/MRAP2 expressing cells was increased, but β-arrestin-2 recruitment was suppressed. MRAP2 did not bias G-protein activation by either receptor, although previous reports show MRAP2 biases MC4R signalling towards Gα_q/11_. The variants did not significantly affect the ability of MRAP2 to modulate MC4R and GHSR signalling. Our results indicate that MRAP2 potentiates the ligand responsiveness of MC4R and GHSR, but has differential effects on β-arrestin-2 recruitment. The MRAP2 variants had no significant effects on the signalling endpoints tested. This suggests that, despite their association with obesity, the variants may be functionally benign, or that the absence of effects reflects limitations inherent to our cellular model. In addition, since MRAP2 can modulate multiple receptors and differentially modulate their signalling, we cannot rule out their influence on body weight regulation via other mechanisms.

## Introduction

Obesity is a complex, multifactorial disease resulting in many co-morbidities, including several types of cancer, depression, cardiovascular diseases and diabetes [1, 2]. Many factors can contribute to obesity, including unhealthy lifestyle (with an important contribution of the environment), psychosocial factors, endocrine factors, use of medication with weight-inducing side effect, as well as genetics [1–3]. Heritability of obesity can range from 40% to 70% and therefore genetics can have a major impact on the development of obesity [2]. Genetic forms of obesity can be classified into two types: monogenic and polygenic [2]. While polygenic obesity, also known as common obesity, is influenced by the environment and involves cumulative effects of many genetic variants, monogenic obesity has a large phenotypic effect caused by a defect in a single gene [2]. The most commonly affected genes causing monogenic obesity are those that encode proteins that are either components or modulators of the leptin-melanocortin pathway in the hypothalamus [2].

Many genes expressed throughout the leptin-melanocortin pathway play important roles in the regulation of body weight through regulation of appetite and/or metabolism [4]. Disruption of these genes, e.g. a pathogenic variant, can cause monogenic obesity [5]. The clinical phenotype of monogenic obesity can include early-onset severe obesity, hyperphagia, and endocrine disorders [6]. Monogenic obesity accounts for over 5% of all cases of severe obesity [5]. Recent studies have identified loss of function (LoF) variants affecting the GPCR accessory protein melanocortin-2 receptor accessory protein-2 (*MRAP2*), which are associated with severe obesity in humans [7, 8]. These individuals also often present with early-onset hyperphagia and/or abnormal eating behaviour, hypertension and hyperglycaemia [9, 10]. *In vivo* experiments confirm the role for MRAP2 in the regulation of body weight: global or brain-specific deletion of *Mrap2* in rodents causes early-onset severe obesity [9, 11, 12]. The early-onset obesity in *Mrap2*-deficient mice preceded hyperphagia, which these mice only developed during adulthood [9]. Despite this, many obesity-associated *MRAP2* variants are reported as likely benign or of uncertain significance due to lack of functional studies or because they have been identified in individuals with normal weight [13].

MRAP2 is a 205 amino acid transmembrane protein expressed mainly in the brain, including the hypothalamus [13, 14]. It was discovered as a homologue of MRAP, which is required for the trafficking and signalling of the melanocortin-2 (adrenocorticotrophic hormone) receptor, a regulator of adrenal development and function [15]. MRAP2 was later characterized as a modulator of cell surface trafficking and signalling of all five melanocortin receptors [14]. In the last decade, studies have shown that MRAP2 modulates the function of multiple GPCRs [12, 14, 16–18]. Although a recently determined MRAP-MC2R cryogenic electron microscopy (cryoEM) structure has given significant insight into how MRAP2 may interact with GPCRs [19], no structural data for these complexes is currently available. However, protein–protein complementation studies as well as bioluminescence resonance energy transfer (BRET)-based techniques show that MRAP2 interacts directly with GPCRs, altering their oligomeric composition and possibly affecting their ability to interact with downstream signalling molecules such as G proteins and β-arrestins [20–22].

The melanocortin-4 receptor (MC4R) and growth hormone secretagogue receptor (GHSR) receptors are co-expressed with MRAP2 in hypothalamic neurons, and have opposing effects on appetite and metabolism. The signalling of both receptors has been shown to be modulated by MRAP2. MC4R, is an anorexigenic receptor in the leptin-melanocortin pathway and pathogenic variants of the *MC4R* gene are the most common cause of monogenic obesity [23, 24]. The similar phenotypic effects of variants in *MRAP2* and *MC4R*, such as early onset obesity and hyperphagia, suggest a functional link. Functional analysis of the effects of *MRAP2* variants on MC4R cAMP signalling has reported a few non-functional MRAP2 proteins, disrupting MC4R signalling and suggesting a causative role in the obesity of the individuals harbouring the variants [13]. However, other studies report contradictory results, for example on MC4R signalling, either potentiating [25, 26], or suppressing canonical Gα_s_/cAMP signalling, and cell surface expression [14, 27]. Moreover, recent studies have shown that MRAP2 might potentiate MC4R signalling through the Gα_q/11_ pathway [28]. GHSR, unlike MC4R, is an orexigenic receptor that promotes food intake and suppresses energy metabolism upon activation by the gut hormone, ghrelin. MRAP2 amplifies GHSR signalling by potentiating ghrelin-induced inositol-1 phosphate (IP1) and suppressing β-arrestin-2 recruitment [12, 22, 29]. However, little is known about the effect of MRAP2 variants on their ability to modulate GHSR signalling. Our hypothesis is that obesity-associated variants of MRAP2 alter their ability to modulate MC4R and GHSR signalling thus providing a mechanistic link to the development of obesity in these patients.

In this study, we identified five *MRAP2* variants in patients with obesity at our Obesity Center CGG (in Dutch: ‘Centrum Gezond Gewicht’), Erasmus MC, Rotterdam, that are either novel (S80F, V91M, and Q174X), have been identified but not functionally characterized (P167A), or have been functionally characterized previously (R125C) on its effect on MC4R signalling. We included the MRAP2 variant K102X as a control in order to validate our methods. K102X has been described as a LoF variant since it does not potentiate α-MSH-induced MC4R cAMP signalling [10]. To explore the impact of these MRAP2 variants on the signalling of receptors involved in appetite suppression (MC4R) and appetite stimulation (GHSR), we analysed the following parameters in HEK293 cells: cell surface expression, total receptor expression, and ligand-induced second messenger responses (α-MSH-induced cAMP response for MC4R and ghrelin-induced Ca^2+^ mobilization for GHSR), β-arrestin-2 recruitment, and alternative Gα_s_ and Gα_q/11_ activation biased signalling.

## Results

### Clinical outcomes

**Figure 1:**
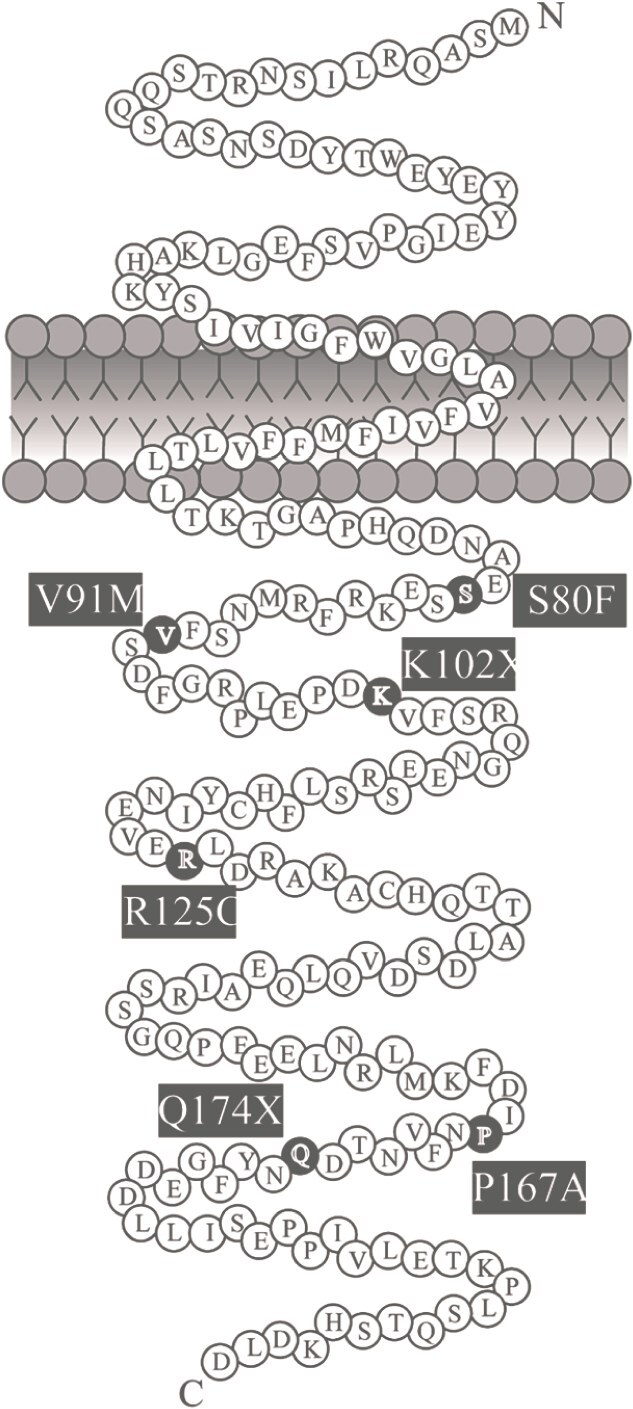
Schematic diagram of MRAP2 (based on the snake diagram from Baron *et al* [10]). The five MRAP2 variants identified in nine patients with obesity are highlighted in black and labelled with the respective amino acid substitution or deletion.

We have identified five heterozygous *MRAP2* variants (S80F, V91M, R125C, P167A, and Q174X) using an obesity gene panel in four paediatric and five adult female patients with severe obesity (Fig. 1 and Table 1) attending the Obesity Center CGG of Erasmus MC in Rotterdam, the Netherlands. The Obesity Center CGG is a tertiary referral center for obesity and also the national center of expertise for genetic obesity, together with the department of Clinical Genetics of Amsterdam University Medical Center. Three unrelated patients were heterozygous carriers of the R125C variant, two related patients were heterozygous carriers of the P167A variant, two unrelated patients were heterozygous carriers of the Q174X variant, and lastly one patient was a heterozygous carrier of the S80F variant (Table 1). Based on literature, S80F, V91M, and Q174X are *novel MRAP2* variants and P167A, although previously identified, has not been assessed functionally. All of the variants were classified as variants of uncertain significance (VUS) based on *in silico* assessment [30]. R125C has been identified and assessed previously for MC4R signalling [10, 28]. None have been assessed for their effects on GHSR function.

**Table 1:** Clinical phenotype of patients with MRAP2 variants (n = 9).

Variant	Nucleotide change	Zygosity	Sex	Adult or child	Nationality	Obesity parents	Variant carried by parent	Weight, kg	Weight SDS	Height, cm	Height SDS	BMI, kg/m^2^	BMI SDS	AoO, years	Hyperphagia	REE (% of predicted)	Fat mass, %
Paediatric patients with MRAP2 variant (n = 4)																	
V91M	271G > A	Het	F	Child	NL	No	n.d.	116.5	5.29	151.9	−2.74	50.49	4.91	4	Yes	96.0	59.3
P167A^*^	499C > G	Het	F	Child	NL	Both	Fa	64.1	4.6	141.6	2.18	31.94	4.37	2	Yes	91.4	n.d.
P167A^*^	499C > G	Het	F	Child	NL	Both	Fa	32.5	4.11	114.8	1.10	24.66	4.20	2	Yes	103.0	n.d.
Q174X	520C > T	Het	F	Child	n.d.	Both	Fa	126.7	5.25	187.2	3.23	36.14	3.71	1	Yes	106.0	48.0
Adult patients with MRAP2 variant (n = 5)																	
S80F	239C > T	Het	F	Adult	NL	Mo	Fa	119.5	n.d.	153.2	n.d.	50.92	n.d.	6	No	n.d.	48.7
R125C	373C > T	Het	F	Adult	NL	Both	n.d.	115.3	n.d.	169.5	n.d.	40.13	n.d.	5	Yes	n.d.	n.d.
R125C	373C > T	Het	F	Adult	NL	No	n.d.	96.5	n.d.	165.5	n.d.	35.23	n.d.	20	No	n.d.	n.d.
R125C	373C > T	Het	F	Adult	NL	Fa	n.d.	148.3	n.d.	180.9	n.d.	45.32	n.d.	7	No	86.0	50.4
Q174X	520C > T	Het	F	Adult	NL	Fa	Fa	138.8	n.d.	170.0	n.d.	48.03	n.d.	6	No	n.d.	n.d.

Abbreviations: AoO, age of onset; Het, heterozygous; BMI, body mass index; F, female; Mo, mother; Fa, father; MRAP2, melanocortin-2 receptor accessory protein 2; NL, Dutch nationality; n.d, not determined; REE, resting energy expenditure; ^*^ = siblings

The BMI of the adult patients ranged from 35.23 to 50.92 kg/m^2^. The BMI SDS of the paediatric patients ranged between +3.71 and + 4.37 (BMI ranging between 24.66 and 36.14 kg/m^2^). The age of onset of obesity ranged from 1.0 to 4.0 years of age for the paediatric patients and from 5.0 to 20.0 years of age for the adult patients. The paediatric patients exhibited hyperphagia while the adult patients did not. It should be noted that the two paediatric siblings with the *MRAP2* variant P167A also were heterozygous carriers of a pro-opiomelanocortin (*POMC*) variant. This variant, E214G (c.641A > G), is classified as benign and is unlikely to be associated with their obesity [31]. For the other paediatric patients, no other abnormalities were detected by SNP array. Five patients (with *MRAP2* variants S80F, R125C, P167A, and Q174X) have inherited their variants from their fathers, of which the majority had obesity. The father of the siblings with the *MRAP2* variant P167A had hyperphagia and early-onset obesity. The patient with *MRAP2* Q174X was not hyperphagic but had early onset obesity and suffered from binge eating disorder since 12 years of age, while the father of this patient also had early onset obesity without hyperphagia. The paediatric patient with *MRAP2* variant V91M showed no hyperphagia, but displayed impaired satiety and satiation. Eight out of nine patients reported obesity-related complications (Table 2).

**Table 2:** Additional characteristic of 9 patients with MRAP2 variants.

Variant	HC, cm	HC SDS	SRR	SRR SDS	DRR	DRR SDS	Heart rate	Other DNA diagnostics	Obesity-related complications	Use of medication	Specific feature of patient
Paediatric patients with MRAP2 variant (n = 4)											
V91M	58.0	1.64	128	>P95	84	>P95	90	n.d.	dyslipidaemia, impaired fasting glucose and impaired glucose tolerance with insulin resistance, vitamin D deficiency	Modafinil, clomipramine, xyrem	Narcolepsy with cataplexies, PCOS
P167A	55.7	2.54	119	P95-P99	70	P50	n.d.	SNP array showed no abnormalities	mild vitamin D deficiency	Vitamin D	Autism, special education due to behavioural problems, red hair, hip dysplasia, scoliosis surgical correction, premature thelarche and pubarche, early closing growth plates
P167A	52.5	1.25	96	P50-P90	59	P50-P90	n.d.	SNP array showed no abnormalities	None	None	Dysplasia of hip, red hair, scoliosis
Q174X	57.0	1.36	122	P50-P90	56	<P50	80	SNP array showed no abnormalities	mild vitamin D deficiency	Vitamin D	Stature deviation feet (rotated inside), increased height, dyslexia
Adult patients with MRAP2 variant (n = 5)											
S80F	56.5	0.5	136	n.d.	81	n.d.	89	n.d.	hypertension, vitamin D deficiency	antihypertensive, Vitamin D, omeprazole, desloratadine	Short stature, ineffective gastric banding, short fingers, sandal gap
R125C	58.0	1.6	115	n.d.	71	n.d.	n.d.	n.d.	OSAS	Fluticasone furoate, budesonide and formoterol fumarate dihydrate	None
R125C	n.d.	n.d.	161	n.d.	87	n.d.	n.d.	n.d.	hypertension	Salbutamol	Lipoedema, red hair, myopia −4 both eyes
R125C	n.d.	n.d.	146	n.d.	86	n.d.	57	n.d.	vitamin D deficiency, painful joints due to obesity	Beclometasone dipropionate, salbutamol, Vitamin D, pantoprazole, ursodeoxycholic acid, metoclopramide	Delayed development as child, only one in family with obesity, binge eating
Q174X	n.d.	n.d.	136	n.d.	73	n.d.	n.d.	n.d.	vitamin D deficiency	Vitamin D	None

Abbreviations: HC = head circumference; SRR = systolic blood pressure; DRR = diastolic blood pressure; n.d. = not determined; OSAS = Obstructive Sleep Apnea Syndrome; PCOS = polycystic ovarian syndrome

### Functional analysis

#### Expression of wild type (WT) and variant MRAP2 proteins

**Figure 2:**
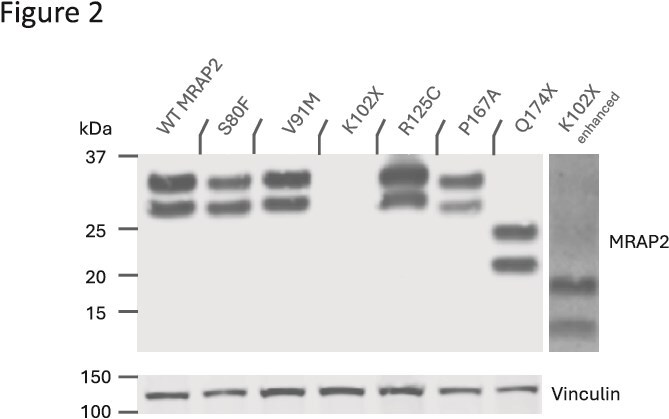
Expression of C-terminally FLAG-tagged WT and variant MRAP2 proteins from lysates of transiently transfected HEK293 cells. Due to significantly low levels of expression, the brightness of the protein band of the K102X variant (K102X enhanced) was amplified to show it is expressed in HEK293 cells. Protein expression of vinculin is shown as control.

Western analysis of C-terminally FLAG-tagged variant MRAP2 shows that variants S80F, V91M, R125C, P167A and Q174X are expressed at similar levels to WT MRAP2 (Fig. 2). Variant Q174X, introduces a premature STOP codon that effectively removes 32 amino acids from the cytoplasmic C-terminus, thus having a lower molecular weight than the other variants. For experimental purposes, we also included a known LoF MRAP2 variant, the previously described MRAP2 variant K102X, which we used as a positive control for LoF in its effect on MC4R signalling [10]. Like Q174X, this variant introduces a premature STOP codon resulting in a C-terminally truncated protein that is only 101 amino acids long (compared with 205 for WT MRAP2), but retains its trans-membrane domain (TMD). This variant is expressed at markedly lower levels likely explaining some of its loss-of function properties. None of the variants affected N-terminal glycosylation, all being expressed in two glycosylated forms.

#### MRAP2 potentiates ligand-induced canonical signalling of MC4R and GHSR

##### MRAP2 potentiates α-MSH efficacy and potency at MC4R cAMP signalling

**Figure 3:**
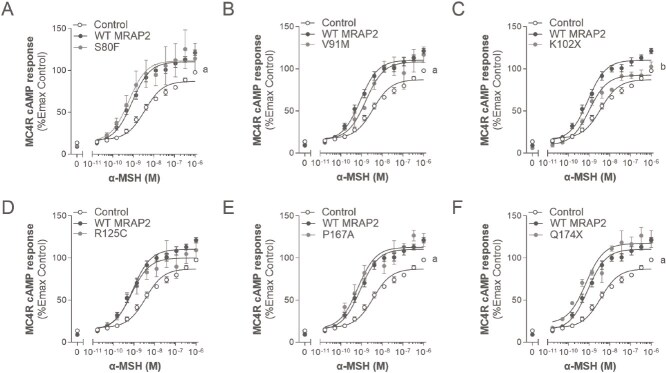
Effects of WT or variant MRAP2 on α-MSH-induced canonical signalling of MC4R. A-F, dose response curves for MC4R in the absence (control), presence of WT MRAP2 and variant MRAP2 to α-MSH, respectively (expressed as %Emax of control). Values represent mean ± SEM of 3 to 4 independent experiments performed in duplicate. Statistical analyses were performed using one-way ANOVA in which each variant was compared separately with control and WT MRAP2 (*a, P* < 0.05 control versus MRAP2; *b, P* < 0.05 WT MRAP2 versus MRAP2 variants).

To determine the impact of WT and variant MRAP2 on MC4R signalling, we assessed the canonical Gα_s_/cAMP pathway upon α-MSH stimulation (Fig. 3 and Table 3). In the presence of WT MRAP2, the maximal cAMP response was increased to 124.4 ± 2.6% compared to control (*P* < 0.001; Fig. 3). Likewise, co-expression of the S80F, P167A and Q174X MRAP2 variants significantly potentiated cAMP responses to α-MSH similarly to WT MRAP2 (Emax 141.2 ± 25.3%, 127.2 ± 5.9%, and 129.5 ± 8.0%, respectively; *P* ≥ 0.05; Fig. 3A, E, F). Unlike the other MRAP2 variants, R125C did not significantly potentiate the cAMP response to α-MSH relative to WT MRAP2 or control, indicating an intermediate effect of the variant (Emax 114.5 ± 15.9%; *P* > 0.05; Fig. 3D). K102X was the only variant showing significant LoF relative to WT MRAP2, likely due its lower expression levels (Emax 106.7 ± 4.1%; *P* < 0.002; Fig. 3C). MRAP2 also increased the potency of α-MSH at MC4R (decreased EC_50_) compared to control (*P* < 0.01). Although this was only matched statistically by V91M (*P* < 0.05; Table 3), all other variants appear to decrease the EC_50_ similarly to WT MRAP2 (*P* > 0.05; Fig. 3 and Table 3).

**Table 3:** MC4R α-MSH-induced cAMP response in the presence of WT and variant MRAP2.

Condition	Emax±SEM (%)	Compared to Control^a^	Compared to WT MRAP2^a^	EC_50_ ± SEM (nM)	Compared to Control^a^	Compared to WT MRAP2^a^
Control	100.0	-	-	2.7 ± 0.6	-	-
WT MRAP2	124.4 ± 2.6	^*^	-	0.7 ± 0.2	^**^	-
S80F	141.2 ± 25.3	^*^	ns	1.1 ± 0.7	ns	ns
V91M	118.6 ± 8.3	^*^	ns	0.2 ± 0.02	^*^	ns
K102X	106.7 ± 4.1	ns	^**^	1.1 ± 0.4	ns	ns
R125C	114.5 ± 15.9	ns	ns	0.9 ± 0.3	ns	ns
P167A	127.2 ± 5.9	^**^	ns	0.7 ± 0.2	ns	ns
Q174X	129.5 ± 8.0	^**^	ns	0.6 ± 0.2	ns (0.06)	ns

Abbreviations: α-MSH = melanocyte stimulating hormone; WT = wild type; n.d. = not determined. Emax was calculated relative to control. The mean of EC50 values was analysed in GraphPad Prism 9 using non-linear curve fitting of the dose response data in ±3 independent experiments.

^a^p-value from one-way ANOVA analysing control, WT MRAP2, and variant MRAP2: ns (not significant): > 0.05 ^*^ < 0.05; ^**^ < 0.01; ^***^ < 0.001

##### MRAP2 potentiates ghrelin-induced GHSR Ca^2+^ mobilization

**Figure 4:**
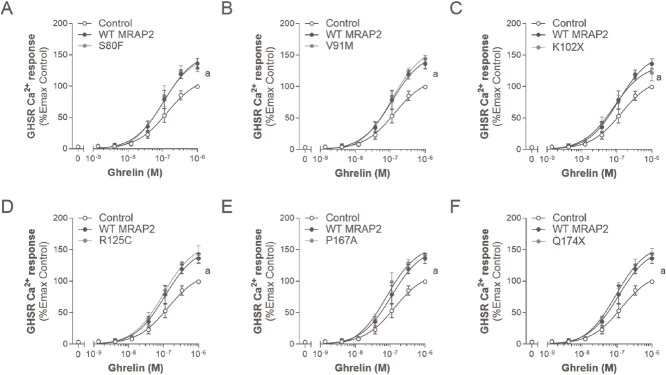
Effects of WT or variant MRAP2 on ghrelin-induced canonical signalling of GHSR. A-F, dose response curves for GHSR in the absence (control), presence of WT MRAP2 and variant MRAP2 to ghrelin, respectively (expressed as %Emax of control). Values represent mean ± SEM of 3 to 4 independent experiments performed in triplicate. Statistical analyses were performed using one-way ANOVA in which each variant was compared separately with control and WT MRAP2 (*a, P* < 0.05 control versus MRAP2; *b, P* < 0.05 WT MRAP2 versus MRAP2 variants).

Next, we assessed the impact of WT and variant MRAP2 on GHSR signalling by measuring the canonical Gα_q/11_/Ca^2+^ mobilization pathway upon ghrelin stimulation (Fig. 4 and Table 4). WT MRAP2 potentiated ghrelin-induced GHSR Ca^2+^ mobilization to 136.2 ± 8.1% (*P* < 0.05), as has been described previously [12]. Likewise, co-expression of the S80F, V91M, R125C, P167A and Q174X variants significantly potentiated Ca^2+^ mobilization (Emax 131.9 ± 7.4%, 144.4 ± 5.2%, 144.3 ± 12.1%, 140.9 ± 2.8%, and 143.2 ± 8.8%; *P* < 0.05) and did not differ from WT MRAP2 in their ability to do this (*P* > 0.05; Fig. 4A, B, D-F). Co-expression of the previously described LoF MRAP2 variant K102X for MC4R signalling potentiated GHSR signalling similarly to that of GHSR/MRAP2 (Emax 128.7 ± 6.0%; *P* < 0.05; Fig. 4C). Unlike α-MSH at MC4R, the potency of ghrelin at GHSR was not significantly altered by co-expression with WT MRAP2 (*P* > 0.05; Table 4). There were no statistical differences in potency between MRAP2 variants and WT MRAP2 (*P* > 0.05).

**Table 4:** GHSR ghrelin-induced Ca^2+^ response in the presence of WT and variant MRAP2.

Condition	Emax±SEM (%)	Compared to Control^a^	Compared to WT MRAP2^a^	EC_50_ ± SEM (nM)	Compared to Control^a^	Compared to WT MRAP2^a^
Control	100.0	-	-	164.3 ± 55.3	-	-
WT MRAP2	136.2 ± 8.1	^*^	-	127.8 ± 48.3	ns	-
S80F	131.9 ± 7.4	^*^	ns	126.4 ± 57.1	ns	ns
V91M	144.4 ± 5.2	^**^	ns	123.2 ± 33.0	ns	ns
K102X	128.7 ± 6.0	^*^	ns	106.7 ± 50.2	ns	ns
R125C	144.3 ± 12.1	^*^	ns	112.2 ± 46.6	ns	ns
P167A	140.9 ± 2.8	^**^	ns	96.3 ± 39.6	ns	ns
Q174X	143.2 ± 8.8	^*^	ns	92.7 ± 14.2	ns	ns

Abbreviations: WT = wild type; n.d. = not determined. Emax was calculated relative to control. The mean of EC50 values was analysed in GraphPad Prism 9 using non-linear curve fitting of the dose response data in ±3 independent experiments.

^a^p-value from one-way ANOVA analysing control, WT MRAP2, and variant MRAP2: ns (not significant): > 0.05 ^*^ < 0.05; ^**^ < 0.01

#### MRAP2 has no biased effect on alternative G protein activation of MC4R and GHSR

**Figure 5:**
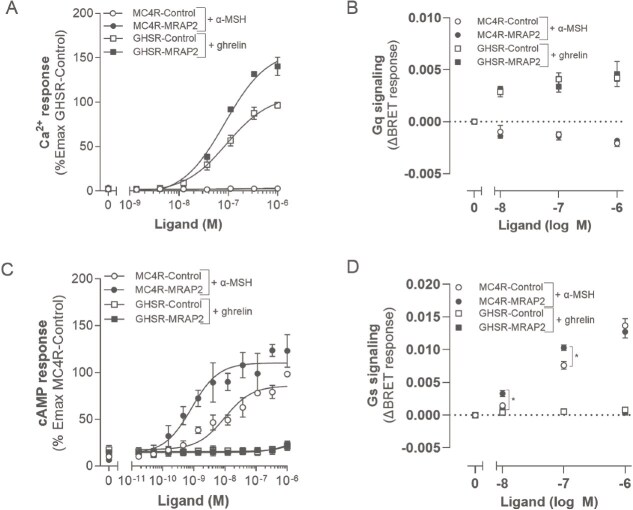
Effects of ligand-induced biased signalling of MC4R towards Gα_q_/Ca^2+^ mobilization and Gα_s_/cAMP response of GHSR in the absence and presence of WT MRAP2 in response to different ligand concentrations. A, lack of Ca^2+^ mobilization response to α-MSH by MC4R either in the presence or absence of MRAP2. GHSR in the absence and presence of WT MRAP2 was used as a positive control. B, Gα_q/11_ biosensor activation upon ligand stimulation (0, 10, 100, and 1000 nM concentrations of both ligands). C, lack of cAMP response to ghrelin by GHSR either in the presence or absence of MRAP2. MC4R in the absence and presence of WT MRAP2 was used as a positive control. D, Gα_s_ activation upon ligand stimulation (0, 10, 100, and 1000 nM concentrations of both ligands). Values represent mean ± SEM of 3 to 4 independent experiments performed in triplicate (^*^, P < 0.05 control versus WT MRAP2).

Previous studies suggest that WT MRAP2 biases signalling of MC4R towards Gα_q_/Ca^2+^ mobilization [28]. Here, we assessed the effect of MRAP2 on specificity of signalling pathway use (cAMP response versus Ca^2+^ mobilization) and G protein activation by MC4R and GHSR using the GloSensor cAMP and aequorin assays, and Gα_s_ and Gα_q/11_ ONE-GO NanoBRET biosensor assays, respectively (Fig. 5) [32]. The ONE-GO NanoBRET biosensor assay measures GPCR activity through the direct activation of specific G proteins upon ligand stimulation [32]. In our cells, WT MRAP2 did not induce MC4R to signal through the Gα_q/11_/Ca^2+^ mobilization pathway upon α-MSH stimulation (Fig. 5A). This result was validated using the Gα_q/11_ ONE-GO biosensor assay with a range of α-MSH concentrations (Fig. 5B). Likewise, GHSR showed no ghrelin-induced Gα_s_/cAMP signalling either in the absence or presence of WT MRAP2 (Fig. 5C). The absence of ghrelin-induced Gαs/cAMP signalling for GHSR was confirmed using the Gα_s_ ONE-GO biosensor assay, showing that MC4R signals through Gα_s_ in the presence and absence of WT MRAP2 while GHSR showed no activation of the Gα_s_ protein by a range of ghrelin concentrations up to 1000 nM; *P* > 0.05; Fig. 5D).

As shown earlier, WT MRAP2 potentiated the cAMP response of MC4R compared to control (Fig. 3 and Fig. 5C). Likewise, MRAP2 potentiated the activation of the Gα_s_ protein upon α-MSH stimulation significantly compared to MC4R-Control at 10 and 100 nM α-MSH (*P* < 0.05; Fig. 5D). The potentiating effect of MRAP2 on Gα_s_ protein activation is lost at a 1000 nM α-MSH concentration, suggesting possible saturation of the response of the biosensor. Unlike MC4R, WT MRAP2 did not potentiate ligand-induced GHSR activation of Gα_q/11_ at any of the concentrations of ghrelin tested (Fig. 5B).

#### Opposing effects of MRAP2 on ligand-induced β-arrestin-2 recruitment by MC4R and GHSR

##### MRAP2 increases α-MSH-induced MC4R β-arrestin-2 recruitment

**Figure 6:**
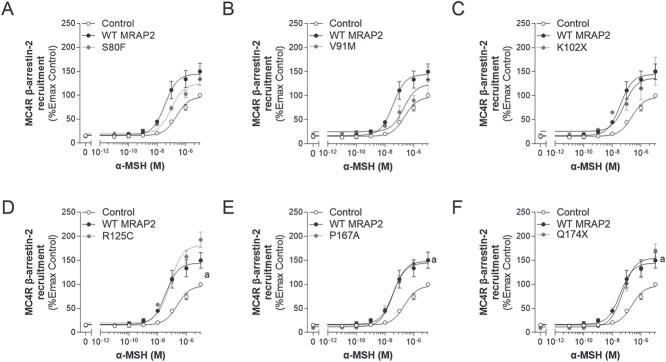
Effects of WT or variant MRAP2 on α-MSH-induced β-arrestin-2 (ARRB2) recruitment to MC4R. A-F, dose response curves for MC4R in the absence (control), presence of WT and variant MRAP2 to α-MSH, respectively (expressed as %Emax of control). Values represent mean ± SEM of 3 to 4 independent experiments performed in duplicate. Statistical analyses were performed using one-way ANOVA in which each variant was compared separately with control and WT MRAP2 (*a, P* < 0.05 control versus MRAP2; *b, P* < 0.05 WT MRAP2 versus MRAP2 variants).

β-arrestin-2 recruitment has been shown to regulate MC4R signalling by inducing receptor internalization as well as non-canonical signalling through the MAPK pathway [33]. We therefore assessed the impact of WT and variant MRAP2 on α-MSH-induced β-arrestin-2 recruitment to MC4R (Fig. 6 and Table 5). WT MRAP2 potentiated ligand-induced β-arrestin-2 recruitment to 155.1 ± 12.8% (*P* < 0.05). While the MRAP2 variants also showed an increase in cAMP response, only P167A and Q174X potentiated MC4R β-arrestin-2 recruitment significantly (Emax 150.9 ± 17.4% and 170.3 ± 13.6% respectively; *P* < 0.05) to similar levels of WT MRAP2 (Fig. 6D and E). K102X tended to show a similar effect (151.2 ± 28.9%; *P* = 0.06; Fig. 6C). The R125C variant showed a slightly greater potentiation of β-arrestin-2 recruitment compared to WT MRAP2 (Emax 192.8 ± 16.2%; *P* = 0.09; Fig. 6D). S80F and V91M showed an intermediate response between control and WT MRAP2, with a trend towards potentiating β-arrestin-2 recruitment similar to WT MRAP2 (Emax 133.4 ± 12.9% and 133.0 ± 27.5% respectively; *P* = 0.09 and ≥ 0.05 respectively; Fig. 6A and B). WT MRAP2 caused a significant decrease in EC_50_ compared to control (*P* < 0.05; Table 5). K102X, R125C, P167A, and Q174X were not different in their impact from WT MRAP2 (*P* ≥ 0.05; Table 5). S80F displayed an intermediate EC_50_ between control and WT MRAP2 (*P* ≥ 0.05). Only V91M caused a significant ~ 10-fold increase in EC_50_ compare to WT MRAP2 (*P* < 0.05), indicating an LoF for this specific endpoint (Table 5). The EC_50_ of S80F and Q174X were not significantly different from control (*P* > 0.05; Table 5).

**Table 5:** MC4R α-MSH-induced β-arrestin-2 recruitment in the presence of WT and variant MRAP2.

Condition	Emax±SEM (%)	Compared to Control^a^	Compared to WT MRAP2^a^	EC_50_ ± SEM (nM)	Compared to Control^a^	Compared to WT MRAP2^a^
Control	100.0	-	-	247.2 ± 64.6	-	-
WT MRAP2	155.1 ± 12.8	^*^	-	35.6 ± 7.1	^*^	-
S80F	133.4 ± 12.9	ns (0.09)	ns	129.7 ± 23.6	ns	ns
V91M	133.0 ± 27.5	ns	ns	449.0 ± 222.3	ns	^*^
K102X	151.2 ± 28.9	ns (0.06)	ns	55.9 ± 28.3	^*^	ns
R125C	192.8 ± 16.2	^***^	ns	49.0 ± 14.9	^*^	ns
P167A	150.9 ± 17.4	^*^	ns	55.7 ± 20.7	^*^	ns
Q174X	170.3 ± 13.6	^**^	ns	67.8 ± 22.9	ns (0.06)	ns

Abbreviations: α-MSH = melanocyte stimulating hormone; WT = wild type; n.d. = not determined. Emax was calculated relative to control. The mean of EC50 values was analysed in GraphPad Prism 9 using non-linear curve fitting of the dose response data in ±3 independent experiments.

^a^p-value from one-way ANOVA analysing control, WT MRAP2, and variant MRAP2: ns (not significant): > 0.05 ^*^ < 0.05; ^**^ < 0.01; ^***^ < 0.001

##### MRAP2 suppresses ligand-induced β-arrestin-2 recruitment by GHSR

**Figure 7:**
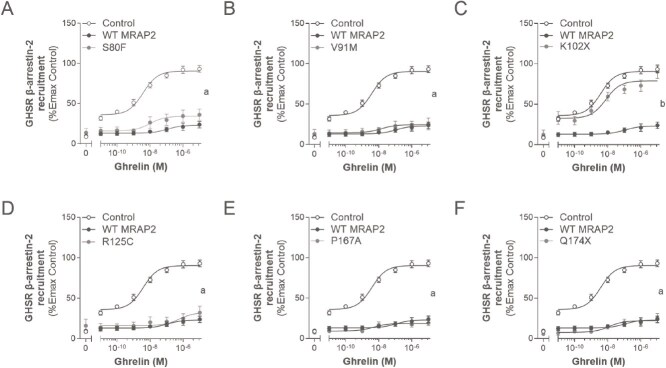
Effects of WT or variant MRAP2 on ghrelin-induced β-arrestin-2 (ARRB2) recruitment to GHSR. A-F, dose response curves for GHSR in the absence (control), presence of WT and variant MRAP2 to ghrelin, respectively (expressed as %Emax of control). Values represent mean ± SEM of 3 to 4 independent experiments performed in duplicate. Statistical analyses were performed using one-way ANOVA in which each variant was compared separately with control and WT MRAP2 (*a, P* < 0.05 control versus MRAP2; *b, P* < 0.05 WT MRAP2 versus MRAP2 variants).

Unlike the potentiated response of α-MSH-induced β-arrestin-2 recruitment to MC4R in the presence of WT MRAP2, MRAP2 significantly reduced the recruitment of β-arrestin-2 to GHSR to 22.1 ± 3.7% (*P* < 0.001; Fig. 7 and Table 6). The MRAP2 variants (S80F, V91M, R125C, P167A, and Q174X) behaved similarly to WT MRAP2 (maximal responses of 36.0 ± 6.9%, 25.7 ± 7.3%, 32.2 ± 8.0%, 18.8 ± 1.9%, and 27.8 ± 4.3% respectively; *P* < 0.001; Fig. 7A, B, D-F). Interestingly, the MRAP2 variant K102X previously analysed for MC4R signalling only, completely lacked the ability to suppress ghrelin-induced β-arrestin-2 recruitment (Emax 90.4 ± 8.5%; *P* > 0.05) as observed for WT MRAP2 (Fig. 7C). The EC_50_ was not significantly altered in the presence of WT or variant MRAP2 (Table 6), although, apart from the control and K102X variant, this was difficult to accurately assess because of the marked suppression of recruitment (Table 6).

**Table 6:** GHSR ghrelin-induced β-arrestin-2 recruitment in the presence of WT and variant MRAP2.

Condition	Emax±SEM (%)	Compared to Control^a^	Compared to WT MRAP2^a^	EC_50_ ± SEM (nM)	Compared to Control^a^	Compared to WT MRAP2^a^
Control	100.0	-	-	3.9 ± 2.2	-	-
WT MRAP2	22.1 ± 3.7	^****^	-	323.5 ± 238.5	ns	-
S80F	36.0 ± 6.9	^****^	ns	10.8 ± 3.2	ns	ns
V91M	25.7 ± 7.3	^****^	ns	18.9 ± 15.6	ns	ns
K102X	90.4 ± 8.5	ns	^****^	8.8 ± 2.6	ns	ns
R125C	32.2 ± 8.0	^****^	ns	406.5 ± 266.6	ns	ns
P167A	18.8 ± 1.9	^****^	ns	5.9 ± 1.6	ns	ns
Q174X	27.8 ± 4.3	^****^	ns	437.1 ± 425.5	ns	ns

Abbreviations: WT = wild type; n.d. = not determined. Emax was calculated relative to control. The mean of EC50 values was analysed in GraphPad Prism 9 using non-linear curve fitting of the dose response data in ±3 independent experiments.

^a^p-value from one-way ANOVA analysing control, WT MRAP2, and variant MRAP2: ^*^ < 0.05; ^**^ < 0.01; ^***^ < 0.001; ^***^ < 0.0001

#### MRAP2 affects cell surface and total expression of MC4R and GHSR differently

##### MRAP2 decreases MC4R cell surface expression

**Figure 8:**
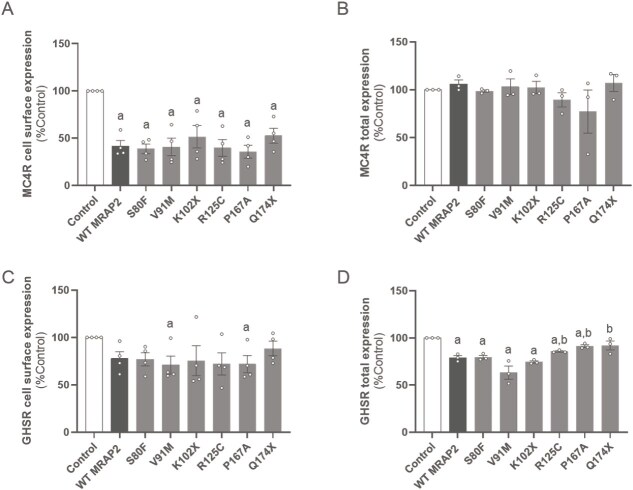
Effects of WT or variant MRAP2 on cell surface and total expression levels of MC4R and GHSR. a, cell surface levels of MC4R in the absence (control) or presence of WT or variant MRAP2. B, total expression levels of MC4R in the absence (control) or presence of WT or variant MRAP2. C, cell surface levels of GHSR in the absence (control) or presence of WT or variant MRAP2. D, total expression levels of GHSR in the absence (control) or presence of WT or variant MRAP2. Values represent mean ± SEM from 3 to 4 independent experiments performed in triplicates. Statistical analyses were performed using one-way ANOVA in which each variant was compared separately with control and WT MRAP2 (*a, P* < 0.05 control versus MRAP2; *b, P* < 0.05 WT MRAP2 versus MRAP2 variants).

Since MRAP2 can affect anterograde trafficking of GPCRs to the cell surface, we assessed the effects of WT or variant MRAP2 on cell surface and total expression of MC4R at basal state (Fig. 8A and B). Although WT MRAP2 increased α-MSH-induced cAMP response, it significantly decreased basal cell surface expression levels of MC4R to 41.8 ± 5.8% compared to control (*P* < 0.0001), which is in line with the increase in β-arrestin-2 recruitment described above. The S80F, V91M, K102X, R125C, P167A and Q174X variants, like WT MRAP2, caused a significant decrease in MC4R cell surface expression (cell surface expression levels at 38.8 ± 5.0%, 40.8 ± 9.2%, 51.6 ± 11.8%, 39.8 ± 8.8%, 35.8 ± 6.8%, and 52.7 ± 7.8% respectively; *P* < 0.05; Fig. 8A). The total expression of MC4R was unaffected by co-expression with WT or variant MRAP2 (Fig. 8B), indicating suppressed trafficking or increased turnover at the cell surface.

##### MRAP2 has little effect on GHSR cell surface expression

We also measured the cell surface expression and total expression of GHSR in the presence of WT or variant MRAP2 at basal state (Fig. 8C and D). Although WT and variant MRAP2 potentiated GHSR Ca^2+^ mobilization and quenched β-arrestin-2 recruitment to GHSR, cell surface expression of GHSR was not affected by the presence of WT MRAP2 (*P* > 0.05), and only the V91M and P167A variants caused significantly decreased cell surface levels compared to control (70.9 ± 9.5% and 71.9 ± 8.9% respectively; *P* ≤ 0.05; Fig. 8C). MRAP2 variants S80F and R125C showed a trend towards decreasing GHSR cell surface expression compared to control (77.1 ± 6.9% and 72.3 ± 11.7% respectively; *P* = 0.05 and 0.08 respectively; Fig. 8C). The total level of expression of GHSR was significantly decreased by WT MRAP2 and the MRAP2 variants S80F, V91M, K102X, R125C, and P167A compared to control (79.1 ± 2.2%, 79.7 ± 1.9%, 63.2 ± 6.9%, 74.5 ± 1.2%, 85.7 ± 0.76%, and 91.3 ± 1.4%, respectively; p < 0.05; Fig. 8D). MRAP2 variants R125C and P167A, however, did not reduce GHSR expression to the same extent as WT MRAP2 (*P* ≤ 0.05), displaying an intermediate phenotype between WT MRAP2 and control conditions. Only the Q174X variant did not affect GHSR expression compared to control (92.0 ± 4.6%; *P* > 0.05), while allowing a greater level of GHSR expression than WT MRAP2 (*P* = 0.05).

## Discussion

**Figure 9:**
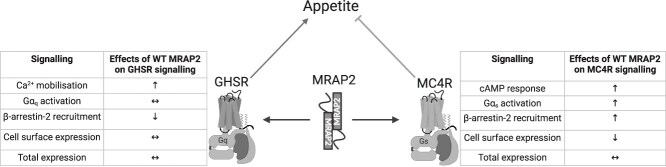
Summary of the effects of WT MRAP2 on GHSR and MC4R signalling, respectively, based on the findings of this study.

In this study we assessed the effect of five obesity-associated MRAP2 variants on their ability to modulate MC4R and GHSR signalling. We also assessed the effects of WT MRAP2 on MC4R and GHSR signalling, as summarised in Fig. 9. MC4R and GHSR are co-expressed with MRAP2 in hypothalamic neurons, and their signalling is modulated by MRAP2 *in vitro* [12, 34]. The *MRAP2* variants were identified in patients referred to the outpatient clinic at our Obesity Center CGG. The gene defect was identified using an obesity gene panel. Apart from the patients with the P167A variant that also carry a benign *POMC* variant, no other obesity associated gene variants were identified in these patients using the obesity panel. Three variants (S80F, V91M, and Q174X) are *novel*, one variant (P167A) has been categorized as a variant of unknown significance (VUS) but not functionally characterized, and one variant (R125C) has been functionally characterized for MC4R signalling only in two previous studies as a LoF or WT-like variant [10, 28]. Our results indicate that these obesity-associated MRAP2 variants affect MC4R and GHSR signalling to a similar degree as WT MRAP2.

Overall, our findings support multiple studies that show an enhancement of ligand-induced MC4R Gα_s_/cAMP signalling in the presence of MRAP2 [9, 21, 26, 28]. The majority of MRAP2 variants analysed in our study (S80F, P167A, and Q174X) potentiated the MC4R cAMP response to a similar degree as WT MRAP2, whereas R125C lacked this effect. Inconsistent results have been reported for the R125C variant, with one study demonstrating LoF [10], and two other studies characterizing this variant as WT-like for MC4R cAMP signalling, which is in agreement with our findings [26, 35]. Our results also confirmed that the K102X variant acts as a LoF variant with respect to cAMP signalling through MC4R, as previously reported [10]. However, the pathogenic effect of this variant in MC4R signalling is most likely due to low expression levels, as K102X showed markedly lower levels of expression compared to WT MRAP2 in transiently transfected HEK293 cells. Like MC4R, GHSR signalling as measured by Ca^2+^ mobilisation was significantly potentiated by MRAP2. This finding is consistent with previous studies showing that MRAP2 not only potentiates ghrelin-induced calcium signalling but also interacts directly with GHSR [12, 29]. However, the lack of effect on GHSR-driven Gq activation also confirms findings of the earlier study [29], suggesting that the modulatory effects of MRAP2 on the GHSR signalling pathway likely occur downstream of Gq. To our knowledge, this is the first study to investigate the impact of MRAP2 variants on GHSR function. Here, in contrast to our findings for MC4R, variant K102X was a LoF variant for β-arrestin-2 recruitment to the GHSR, while significantly increasing the ability of MRAP2 to potentiate ghrelin-induced Ca^2+^ mobilization, despite its low levels of expression. Similarly, R125C appeared to have partially lost the ability to potentiate MC4R-cAMP signalling, but did not affect GHSR function. It should be pointed out that we observed a significant effect on Ca^2+^ mobilization using a GHSR:MRAP2 ratio of 1:1, whereas previous studies suggest that a 1:5 ratio is required to detect potentiation of an IP1 response [29]. These findings are interesting from a physiological perspective. Theoretically, LoF of MC4R signalling would enhance appetite, especially when the orexigenic signalling of ghrelin at GHSR remains intact. Indeed, mice in which MRAP2 is knocked out specifically in MC4R expressing neurons show a greater increase in body weight (~30%) at 12 weeks of age than full-body MRAP2 knockout mice (~10%) of similar age [9, 12, 34]. The former mouse model would have retained MRAP2-potentiation of GHSR signalling, thereby exaggerating orexigenic signalling; this potentiation of GHSR signalling was absent in the full-body MRAP2 knockouts.

The effects of MRAP2 on β-arrestin-2 recruitment to both GPCRs were analysed in our study. MRAP2 clearly potentiates ligand-induced β-arrestin-2 recruitment by MC4R. Likewise, MRAP2 variants R125C, P167A and Q174X significantly potentiated ligand-induced β-arrestin-2 and MC4R. Given that β-arrestin recruitment is a cellular feedback mechanism that suppresses GPCR activity, it is perhaps counterintuitive that MRAP2 potentiates ligand-induced signalling. However, variants S80F and V91M may partially impair the recruitment of β-arrestin-2 to MC4R, whereas variant K102X behaved similarly to WT MRAP2 for this endpoint. As has been described before, and unlike MC4R, we found that MRAP2 markedly suppresses ligand-induced β-arrestin-2 recruitment to GHSR [22]. However, the majority of the variants we assessed did not modify this effect. While the K102X variant maintained its ability to potentiate β-arrestin-2 recruitment to MC4R, it entirely lost its capacity to suppress β-arrestin recruitment to GHSR. Interestingly, this variant only showed a trend towards potentiated Ca^2+^ mobilization versus control, but did not differ significantly from WT MRAP2. These results suggest that the MRAP2 variant K102X affects MC4R and GHSR signalling in a receptor-specific way. It is intriguing that K102X is not LoF for all signalling endpoints given its low expression levels. This indicates that certain signalling pathways are more sensitive to reduced expression of this variant than others and that this is also receptor specific. It could also indicate that the C-terminus of MRAP2 is more critical for its modulation of GHSR function than for MC4R.

The cell surface expression of MC4R was significantly reduced in the presence of WT MRAP2 and all assessed MRAP2 variants, but total expression levels were comparable to control. This is consistent with a role for MRAP2 in trafficking MC4R to the cell surface and is in line with previous studies [14]. We speculate that MRAP2 modulates not only ligand-mediated, but also constitutive activity of MC4R which could be a potential mechanism explaining the reduction in surface levels in the absence of ligand. Constitutive activity was originally described in in vitro experiments in which AgRP was shown to exhibit inverse agonist properties on basal activity of MC4R [36]. Srinivasan et al. later showed that this constitutive activity was driven and maintained by the N-terminal domain of MC4R which acts as a tethered ligand [37]. Constitutive activity of receptors is known to increase their rate of internalisation [38]. It is possible that MRAP2 modulates the interaction of the N-terminal domain with its binding pocket causing an increased rate of internalisation. MRAP2 also regulates oligomerisation of the receptor [21], which could also affect constitutive internalisation rate. Furthermore, a recent study identified an obesity-associated MRAP2 variant, V91A, which is analogous to the V91M that we describe. Like V91M, V91A had no effect on the ability of MRAP2 to modulate MC4R signalling, including cAMP response, IP3, and cell surface expression and ligand-induced internalization [28]. It was concluded that V91A is unlikely to contribute to the obesity phenotype [28]. While MRAP2 reduced MC4R cell surface expression, we found that it had no effect on GHSR cell surface expression, consistent with previous studies [29]. This fits with the previously described direct effects of MRAP2 on GHSR signalling within the cell, rather than modulating cell surface expression [22, 29]. Of the MRAP2 variants, the majority had no significant effect on cell surface expression suggesting that the variants had no or only minor effects on MRAP2 function with respect to GHSR signalling.

Our findings show that MRAP2 modulates MC4R and GHSR signalling differently, and that the *MRAP2* variants we identified in our patients with obesity mostly behave similarly to WT MRAP2. It should be noted that two patients with the variants Q174X and V91M had early-onset obesity without hyperphagia, which is a similar phenotype to that displayed in *Mrap2*-deficient mice which have early-onset obesity without initial hyperphagia [9]. Furthermore, the patient with the V91M variant also suffered from binge eating disorder from the age of 12 years old. Eating behaviour problems have been reported previously in patients with *MRAP2* variants [10]. However, the paediatric patients with other *MRAP2* variants developed early-onset hyperphagia, which is likely the cause of their early-onset obesity. A previously published study linked LoF MRAP2 variants to patients with hyperphagic obesity displaying hyperglycaemia and hypertension [10]. However, no patterns were identified for blood pressure or REE in our patient population. This lack of effect of the MRAP2 variants relative to WT MRAP2 suggests that they are benign or that other signalling endpoints/parameters need to be investigated. For example, MRAP2 modulates signalling of a range of other appetite-regulatory receptors, such as the orexigenic OXR1 and anorexigenic PKR1 receptors [12, 16]. A recent study also showed that MRAP2 may potentiate ligand-induced MC3R signalling by suppressing β-arrestin-2 recruitment, and thereby increasing internalization [18, 35]. Another recent study showed that MC4R signalling through the Gα_q/11_-IP3 pathway is modulated by MRAP2, and that variants not only impaired Gα_s_-cAMP signalling but also Gα_q/11_-IP3 signalling [28]. Interestingly, this study showed that the R125C MRAP2 variant impaired Gα_q/11_-IP3 signalling and was categorized as a LoF variant, while it did not show any effect relative to MRAP2 on Gα_s_-cAMP signalling. This reaffirms the impact of MRAP2 on intra-cellular signalling pathways originally discovered for GHSR which may be linked to modulation of ligand-induced Gα_q/11_ signalling and β-arrestin recruitment. We show that MRAP2 does not cause biased signalling for MC4R towards Gα_q/11_ in our cells, therefore we could not confirm the modulation of MRAP2 or the effect of the MRAP2 variant on Gα_q/11_ pathway found in a previous study [28]. Furthermore, we show that MRAP2 significantly potentiates activation of Gα_s_ upon α-MSH stimulation, suggesting that MRAP2 may have a direct impact on Gα_s_ function or interaction with MC4R. A preprint investigating the effects of MRAP2 on MC4R signalling supports our findings, showing that in the presence of MRAP2, the maximal response of the Gα_s_ activation is not altered by MRAP2 compared to cells lacking MRAP2. However, MRAP2 seems to lower EC_50_ (increase in potency), indicating potentiation of activation of Gα_s_ signalling [21]. In relation to this, MRAP2 co-expression did not lead to GHSR biased signalling towards the Gα_s_ pathway.

MRAP2, along with MRAP, were shown to interact with all the melanocortin receptors using immunoprecipitation, and modulate signalling of all the receptors [14]. Recent studies have reported a direct interaction between human MRAP2 and human GHSR, as well as feline MRAP2 and feline MC4R, using the NanoBiT protein–protein interaction assay in live cells at basal and upon ligand stimulation [12, 39]. Additionally, the oligomeric equilibrium of MC4R, MC3R and GHSR was shown to be altered and changed to a monomeric state in the presence of MRAP2, disrupting oligomerisation, increase signalling of the receptors, and further indicating an interaction between MRAP2 and the receptors [20, 21]. A homology model between MRAP2 and MC4R was recently developed, showing that the TMD of MRAP2 formed interaction sites with the TMD5, TMD6, and TMD7-ECL3 sites of MC4R. These sites of the receptor are confirmed to be involved in GPCR-GPCR dimer structures [21]. Although research lacks the cryoEM structure of MRAP2 with any GPCR, the recent prediction models have observed similar site-interactions between MRAP2 and MC4R, GHSR, and MC3R to those in a recently solved cryo-EM structure of the MRAP-MC2R complex [19, 21].

While prediction models for pathogenicity, such as Alphafold, have focused on the impact of variants in the transmembrane domain of MRAP2, all variants in this study are located in the C-terminus of MRAP2 (Fig. 1). In line with the location of the variants, functional approaches have demonstrated that the C-terminal region of MRAP2 is critical for the modulation of GHSR signalling [29]. Moreover, four C-terminal MRAP2 variants were demonstrated to disrupt ligand-induced MC4R internalization [28]. Similar observations were made for this region in OX1R and PKR1 [16]. Recently, Ojeda-Naharros and colleagues showed that MRAP2 may be oriented with its C-terminus on the intracellular side of the cell membrane [40]. The observed lack of effect of MRAP2 on Gα-protein signalling for both MC4R and GHSR in this study, suggests that MRAP2 modulates G-protein signalling further downstream. The intracellular localization of the MRAP2 C-terminus would allow this domain of the protein to directly affect these downstream intracellular signalling cascades. This aligns with the LoF of the K102X variant, which lacks a large part of the C-terminus, although the function of this variant is likely compromised by its lower level of expression. If the C-terminus were this important in MRAP2’s effects on GPCR signalling, one would expect—at least some—of the other variants in this study to also affect MRAP2 function. Additionally, the independent identification of two variants of V91 (V91A and V91M) in patients with obesity strongly implies the involvement of this amino acid or region in MRAP2 function [28]. However, such effects were not found in this and other studies that used HEK293 cells as a screening model for these variants [28]. A possible reason for this could be the choice of the *in vitro* model. Part of the mechanism through which MRAP2 regulates MC4R function is by facilitating MC4R’s localization to primary cilia [34, 40]. MRAP2 is specifically required for the entry of MC4R into primary cilia, while the inverse agonist AgRP and β-arrestin seem to be essential for the accumulation and exit of the receptor from the neuronal primary cilium by suppressing its activity [40]. Even though HEK293 cells have been an often-used model for ciliogenesis, only a small portion of these cells (~5%) form primary cilia [41]. Although HEK293 cells are easily cultured and transfected, which facilitates fast and efficient screening for functional effects of variants, their relative deficiency of cilia formation may obscure critical aspects of MC4R biology that depend on ciliary localization and function. We speculate that pathogenic *MRAP2* variants may impair the trafficking of MC4R to neuronal cilia *in vivo*, disrupting the MC4R-dependent regulation of appetite. Although we confirmed that MRAP2 and the variants are expressed in our model system at the protein level, the low frequency of primary cilia in HEK293 cells may mask the full effects of MRAP2 on the aspects of MC4R signalling measured in this study. This, in theory, could affect the functional characterisation of MRAP2 variants and their effect on MC4R signalling. For GHSR, it has been reported that GHSR is not localised in primary cilia although the effect of MRAP2 on trafficking of this receptor to cilia has not been studied [42]. Ideally, the use of cell lines containing primary cilia, the development of human neuronal cell lines or induced pluripotent stem cell-derived neurons would enhance the validity of *in vitro* screening assays, with the latter method providing the opportunity to use patient-derived materials. Until that time, results from studies in other model systems should be carefully interpreted: while a loss of effect indicates pathogenicity, lack of effect of a variant in these model systems do not necessarily render the variant benign.

In conclusion, although associated with obesity, the *novel* MRAP2 variants examined in this study lack any major differential effects relative to MRAP2 in their ability to modulate ligand-induced canonical signalling pathways of MC4R and GHSR. However, since MRAP2 can modulate multiple receptors as well as differentially modulate their signalling pathways, we cannot rule out their influence on body weight regulation via other mechanisms.

## Materials and methods

### Clinical analyses

#### Patient cohort and sequencing analysis

Collection of clinical phenotype and DNA samples from patients were obtained at the Obesity Clinic CGG, Erasmus University Medical Center Rotterdam. Sequencing analyses were performed by the Genome Diagnostics section of the Department of Genetics, University Medical Center Utrecht, the Netherlands and the Department of Human Genetics, Amsterdam University Medical Center, the Netherlands [43]. DNA samples were analysed as previously described [24]. The study involving human participants was reviewed and approved by the Ethical Committee of the Erasmus MC, University Medical Centre, Rotterdam (paediatric study MEC-2012-257 and adult study MEC-2023-0029). Informed consent of all participants and/or caregivers was obtained, when needed, according to the approved protocol.

The *MRAP2* variants were identified using an obesity gene panel conducted at University Medical Center Utrecht and Amsterdam University Medical Center [24, 44]. *MRAP2* variants were classified as described previously [24, 30, 44]. All variants were identified and assessed *in silico* for pathogenicity according to the ACMG guidelines [30].

Resting energy expenditure (REE) was measured and calculated as previously described [24, 45–48]. Body composition in children and adults was measured as previously described [24, 49]. Hyperphagia was assessed by the treating medical specialist, based on indicators such as persistent hunger with/without associated distress, impaired satiation and/or satiety, preoccupation with food, food-seeking behaviour or episodes of secret eating [44].

### Functional analyses

#### Expression constructs and site-directed mutagenesis

The human *MC4R* and *GHSR* expression constructs in pcDNA3.1+ were obtained from the cDNA Resource Center (www.cdna.org, USA). The human MRAP2 cDNA (Sino Biological Europe GmbH, Germany) was cloned into pcDNA3.1(+). To assess the relative expression levels of wild type (WT) and variant MRAP2, plasmids encoding C-terminally FLAG-tagged proteins were used. For the K102X and Q174X truncating variants, the DNA segments from the premature STOP codons up to, but not including, the FLAG-tags were excised using PCR generated plasmids that were assembled *in vivo* using a method described previously [50]. For β-arrestin-2 recruitment NanoBiT-complementation assays MC4R was C-terminally tagged with LgBiT using pBiT2.1-C[TK/LgBiT] (Promega, USA) and co-transfected with human ARRB2 (β-arrestin-2; SinoBiological Europe GmbH, Germany) N-terminally tagged with SmBiT using the pBiT2.1-N[TK/SmBiT] expression plasmid (Promega, USA). Conversely, GHSR was C-terminally tagged with SmBiT using pBiT2.1-C[TK/SmBiT] (Promega, USA) and ARRB2 was N-terminally tagged with LgBiT using pBiT1.1-N[TK/LgBiT] (Promega, USA). For cell surface and total expression NanoBiT complementation assays, the MC4R and GHSR cDNAs were N-terminally tagged with HiBiT by cloning into pBiT3.1-secN [CMV/HiBiT/Blast] (Promega, USA). The orientation of NanoBiT tags on the receptors and MRAP2 was optimised as described in our previously published studies of β-arrestin-2 recruitment by MC4R and GHSR [24, 51]. Site directed mutagenesis was used to generate the *MRAP2* variants as described previously [24]. All constructs were verified by Sanger sequencing. We used the MRAP2 variant K102X as a control in order to validate our methods. K102X has been described as a LoF variant since it does not potentiate a ligand-induced MC4R cAMP response [10].

#### Cell lines and transfection

Human embryonic kidney 293 (HEK293) cells (ECACC Cat# 85120602, RRID:CVCL_0045, UK) were cultured at 37°C in Dulbecco’s modified Eagle medium/F12 (DMEM/F12; GIBCO, USA), 10% heat-inactivated foetal calf serum (FCS), 2.5 mM GlutaMAX, 100 units/ml penicillin and 100 μg/ml streptomycin (P/S) (ThermoFisher Scientific, the Netherlands) under humidified air containing 5% CO2 for all experiments.

#### Analysis of protein expression of MRAP2 WT and variants

To assess the protein expression of MRAP2 WT and variants, cells were seeded at 3 × 10^5^ cells/well in poly-D-lysine-coated 6-well plates. The next day, cells were transiently transfected with 500 ng/well of WT or variant MRAP2 expression plasmid using FuGENE HD (Promega, USA). After 48 h, the cells were washed with PBS (Gibco, USA) and lysed with Pierce RIPA buffer (Thermo Fisher, the Netherlands) supplemented with 1:100 phosphatase inhibitor cocktail 2 (Sigma Aldrich, USA) and 1:100 protease inhibitor (Roche, Germany). Lysates were sonicated for 10 seconds and the supernatant was collected. The protein content was measured using a Bradford assay (BioRad Protein Assay). Samples (20 μg) were run on mPAGE 10% Bis-Tris Precast Gels (Sigma Aldrich, USA) and transferred to nitrocellulose membranes (Cytiva, UK). They were then blocked for one hour at room temperature with PBS containing 3% non-fat milk and tagged MRAP2 was detected using an anti-FLAG antibody (D6W5B Rabbit Monoclonal Antibody #14793; Cell Signalling Technology, USA) in 1:1000 in Intercept Blocking Buffer (Li-Cor, NE, USA) containing 0.1% Tween 20. Membranes were incubated overnight at 4°C and then washed in PBS containing 0.1% Tween 20 before being incubated for one hour in a mixture of anti-rabbit-IRDye 800 CW (1:10000 in PBS, 0.1% Tween 20, 5% non-fat milk). Membranes were also incubated using an anti-vinculin primary antibody (7F9 Mouse Monoclonal Antibody #sc-73 614, Santa Cruz Biotechnology, USA), in 1:1000 in Intercept Blocking Buffer (Li-Cor, NE, USA) containing 0.1% Tween 20, overnight at 4°C, washed thoroughly and incubated for one hour in a mixture of anti-mouse-IRDye 800 CW (1:10000 in PBS, 0.1% Tween 20, 5% non-fat milk). After washing in PBS 0.1% Tween 20, membranes were scanned with an Odyssey CLx (Li-Cor, NE, USA) and were analysed using Image Studio lite (Li-Cor, NE, USA).

#### Measurement of ligand-induced CRE luciferase

Cells (1.3 × 10^6^) were seeded in 25 cm^2^ culture flasks. The next day, cells were transiently transfected with 400 ng of pcDNA3.1(+)-MC4R or GHSR expression plasmid and 400 ng of empty vector (EV) pcDNA3.1(+), WT MRAP2, or variant MRAP2 in a 1:1 ratio, 2.2 μg CRE6-reporter plasmid (Promega, USA), and 1 μg pRL-SV40 plasmid (Promega, USA). Cells (4 × 10^4^ cells/well) were reseeded into a poly-D-lysine (PDL)-coated clear 96 well plate and incubated overnight. Cells were stimulated with α-MSH or ghrelin (Tocris, UK) at concentrations ranging from 10^−11^ to 10^−5^ M for 6 h. The CRE response was measured using the Dual-Glo Luciferase Assay System according to the manufacturer’s protocol (Promega, USA) in a CLARIOstar plus plate reader (BMG Labtech).

#### Measurement of ligand-induced Ca^2+^ mobilization

Cells (1.3 × 10^6^) were seeded to 25 cm^2^ culture flasks. The next day, cells were transfected with 50 ng of GHSR or MC4R, and either 50 ng of empty vector (pcDNA3.1+) or 50 ng WT/variant MRAP2, and 2 μg of mtAequorin plasmid (a gift from Dr L. Barak from Duke University Medical Center, NC, USA). Aequorin protocol was followed as previously described [51]. Then, 50 μl of cells (1 × 10^4^ cells/well) were injected into each wells of a 96-well plate containing a dilution series of either ghrelin or α-MSH and the luminescent signal was measured instantly using a plate reader (CLARIOstar plus, BMG Labtech). Finally, 50 μl of 0.3% Triton X-100 was injected and luminescence was measure in order to measure the total remaining activity of Aequorin. The ligand response was corrected for total luminescence (sum of both ligand and triton measurements), yielding the fractional response.

#### Measurement of Gα_s_ and Gα_q/11_ activation

Cells (3 × 10^5^) were transfected in 800 μl with 50 ng of Gα_s_/Gα_q/11_ ONE-GO plasmid [52], 200 ng of receptor, 200 ng of pcDNA3.1(+) or WT MRAP2. Cells were seeded at 3 × 10^4^ cells/well in PDL-coated opaque white 96-well plates. After 48 h, NanoBRET reagent diluted 1:1000 in HBSS was added to the cells and baseline BRET signal was measured using the CLARIOstar plus reader. Next, cells were stimulated with 1 μM of ligand or a condition without agonist (vehicle), and BRET signal (535 nm fluorescence/460 nm luminescence) was measured every minute for 6 minutes. BRET data was displayed as the difference from BRET signal (535/450 BRET ratio) to vehicle and calculated to % control of the respective receptors.

#### Measurement of β-arrestin-2 recruitment

Cells (2 × 10^4^ cells/well) were seeded on a PDL-coated clear 96 well plate, and for each GPCR, transiently transfected 25 ng of NanoBiT tagged MC4R/GHSR expression plasmid, NanoBiT-tagged β-arrestin-2, and pcDNA3.1(+) empty vector, WT MRAP2 or variant MRAP2 (1:1:1 ratio). NanoBiT protocol was followed as described previously [24]. Next, cells were stimulated with ligand at concentrations ranging from 10^−11^ to 10^−5^ M for GHSR and 10^−11^ to 10^−6^ M for MC4R and measured in the CLARIOstar plus reader.

#### Quantification of extracellular and total expression

Cell surface expression and internalization of MC4R and GHSR were measured using the Nano-Glo HiBiT Extracellular Detection System (Promega, USA), and the total expression of MC4R and GHSR were measured using the Nano-Glo HiBiT Lytic Detection System (Promega, USA). Cells (2 × 10^4^ cells/well) were seeded in a PDL-coated clear bottom white 96-well plate. For each receptor, cells were transiently transfected with 0.25 ng of N-terminally HiBiT-tagged MC4R or GHSR, and 0.25 ng of pcDNA3.1(+) empty vector, and WT or variant MRAP2 (1:1 ratio). HiBiT Extracellular and Lytic Systems were followed as previously described [24].

#### Data analysis

Prism (version 9.0.0 for Windows; RRID: SCR_002798; GraphPad Software, Boston, MA, USA) was used to perform non-linear curve fitting of the dose response data, calculate EC_50_, and perform statistical tests. Differences between controls (cells transfected with receptor expression plasmids together with the empty vector used for MRAP2 expression), WT and variant MRAP2 were assessed using one-way ANOVA and Tukey post-hoc test. Differences between controls and WT MRAP2 in the G-ONE GO assay at each ligand concentration were assessed using t-test. Results were derived from 3–4 independent experiments using duplicate or triplicate samples. Data for MRAP2 variants are expressed relative to empty vector controls (set to 100%).

## Notes

*Conflict of interest statement*: J.V. has received royalties from AMH assays, paid to the institute/laboratory with no personal financial gain. E.v.d.A. has no personal financial gain, she is principal investigator on clinical trials with setmelanotide for patients with leptin-melanocortin pathway defects, for which the Erasmus MC has research contracts with Rhythm Pharmaceuticals. C.J.d.G has been involved in sponsor-initiated trials by Rhythm Pharmaceuticals investigating setmelanotide and has worked with Rhythm Pharmaceuticals on disease awareness lectures. A.R., K.P., F.V., E.v.d.W., E.v.R., M.v.H. and P.D. have no conflicts of interest.
